# Language Variation in the Writing of African American Students: Factors Predicting Reading Achievement

**DOI:** 10.1044/2021_AJSLP-20-00263

**Published:** 2021-11-01

**Authors:** Lisa Fitton, Lakeisha Johnson, Carla Wood, Christopher Schatschneider, Sara A. Hart

**Affiliations:** a Communication Sciences & Disorders Department, University of South Carolina, Columbia; b School of Communication Science & Disorders, Florida State University, Tallahassee; c Department of Psychology, Florida State University, Tallahassee

## Abstract

**Purpose:**

This study aims to examine the predictive relation between measures obtained from African American students' written narrative language samples and reading achievement, as measured by standardized academic assessments.

**Method:**

Written language samples were elicited from 207 African American students in Grades 1–8. The samples were examined for morphosyntactic variations from standardized written Generalized American English (GAE). These variations were categorized as either (a) specific to African American English (AAE) or (b) neutral across AAE and standardized written GAE (i.e., considered ungrammatical both in AAE and in standardized written GAE). Structural equation modeling was employed to then examine the predictive relation between the density of AAE-specific forms in students' writing and their performance on standardized assessments of literacy and reading vocabulary. This relation was examined while accounting for the density of dialect-neutral morphosyntactic forms, reported family income, age, and written sample length.

**Results:**

The written samples were highly variable in terms of morphosyntax. Younger students and those from lower income homes tended to use AAE-specific forms at higher rates. However, the density of AAE-specific forms did not significantly predict standardized literacy scores or reading vocabulary after accounting for dialect-neutral variations, income, and sample length.

**Conclusions:**

These results support the ongoing need to better understand the language, literacy, and overall academic development of students from all backgrounds. It may be essential to focus on dialect-neutral language forms (i.e., morphosyntactic forms that are consistent across both AAE and standardized written GAE) in written samples to maximize assessment validity across students who speak varying dialects of English.

**Supplemental Material:**

https://doi.org/10.23641/asha.16879558

In 2019, only 18% of African American students met criteria for reading proficiency on the National Assessment of Educational Progress (National Center for Education Statistics, 2019). Approximately 11% of African American students met criteria for writing proficiency (National Center for Education Statistics, 2012). The overrepresentation of African American individuals among students performing at below basic levels has received considerable attention in recent years (Gatlin & Wanzek, 2017; Washington et al., 2019). Numerous factors, such as socioeconomic background and variability in the quality of language and literacy environments, have been explored as contributors to this vulnerability for academic underachievement, but no single factor provides a complete explanation (Terry et al., 2018). For example, although African American students are more likely to come from low-income backgrounds than Caucasian students (Reardon et al., 2018), gaps in academic performance exist after controlling for socioeconomic status and school composition (Bohrnstedt et al., 2015; Reardon et al., 2019). Given the multifaceted and structural mechanisms (i.e., systemic racism) that underly achievement gaps (Merolla & Jackson, 2019), there is a need to evaluate predictors of achievement in a more comprehensive framework, to gain a fuller picture of students' experiences and environments, and how they might contribute to literacy development.

The body of literature examining literacy development among African American students includes relatively few studies that have focused specifically on the development of written language skills (Gatlin & Wanzek, 2015; Ivy & Masterson, 2011; Puranik et al., 2019). Writing is a critical skill, not only for general academic learning and annual standardized testing but also as a metric for evaluating ability related to higher education and suitability for employment. Written performance is commonly used to monitor progress in educational settings, and therefore, is of particular interest as a key component of overall academic achievement (National Center for Education Statistics, 2019; Wagner et al., 2011).

For African American students, nonmainstream dialect use has been suggested as a potential explanatory factor for observed achievement gaps (see Siegel, 1999), given that correlations have been observed between nonmainstream dialect use and the development of literacy-related skills, including writing (Gatlin & Wanzek, 2015). Although all individuals speak a dialect, some American dialects other than Generalized American English (GAE; also called Standardized American English or Mainstream American English) have been discriminatorily stigmatized as “inferior” language systems (Baker-Bell, 2020; Brown, 2019; Dovchin, 2020). It is important to emphasize that all dialects are rule-bound systems with no inherent superiority or inferiority. Although it is common to not recognize GAE as a specific dialect (Hamilton et al., 2018), GAE is neither exceptional nor the default dialect of American English (Charity Hudley et al., 2018; Oetting et al., 2016).

Dialects of American English other than GAE have been broadly termed *Nonmainstream American English (NMAE).* African American English (AAE), a dialect most commonly spoken with variable density by African American individuals in the United States, is one such NMAE dialect that has unique morphological, semantic, syntactic, phonological, and pragmatic rules (Baker-Bell, 2020; Stockman, 2010). Some of the morphosyntactic forms of AAE overlap with several other NMAE dialects such as Southern White English (Oetting, 2019) and Gullah/Geechee (Berry & Oetting, 2017). Some of these rules contrast with rules of GAE, whereas other rules of AAE are consistent or neutral to GAE (Charity et al., 2004; Washington & Craig, 2002). In this article, we use the term *dialect-neutral* to refer to morphosyntactic and phonological rules that are consistent across AAE and GAE (Terry, 2014). We use the term *AAE-specific* to refer specifically to rules of AAE that are contrastive with GAE, and *dialect-specific* to refer generally to rules of NMAE dialects that are contrastive with GAE (Stockman, 2010).

Researchers have observed a negative association between African American students' density of AAE-specific forms and their reading development (Craig & Connor et al., 2003; Terry et al., 2016). Much of this literature was synthesized in a 2015 meta-analysis that Gatlin and Wanzek conducted to quantify the relation between the density of nonmainstream dialect-specific forms and literacy development. Findings revealed an overall moderate negative correlation between the density of dialect-specific forms and literacy performance, unmoderated by socioeconomic status or grade level (Gatlin & Wanzek, 2015). Critically, the authors highlighted the need for caution in interpreting these findings as indicative of linguistic interference (i.e., NMAE negatively impacting literacy development). Dialectal variation provides only a single piece of the complete picture, which must be viewed in context to better understand achievement gaps observed between African American and Caucasian students (Gatlin et al., 2016; Terry et al., 2018).

The overall purpose of this article was to evaluate the relation between African American students' density of AAE-specific forms in writing and their reading achievement, while accounting for socioeconomic status and dialect-neutral writing skills. In the following literature review, we summarize work that has examined the relations between dialectal variation and achievement, detail the value of focusing on the density of dialect-specific forms, and describe our approach for examining the written language of African American students.

## Dialect Use and Academic Achievement

Numerous studies have examined the relations between students' dialectal variation and their literacy skills (Kohler et al., 2007; Terry et al., 2012, 2016). In considering this body of literature, it is essential first to review how dialectal variation has generally been operationalized. Many studies quantify dialectal variation by counting how often dialect-specific forms appear in samples of students' language and then computing a token-based measure of frequency of occurrence, or “dialect density” (Horton-Ikard & Miller, 2004; Washington & Craig, 2002). Three widely used token-based measures of dialect density, which were reviewed by Oetting and McDonald (2002), account for both the number of dialect-specific forms observed and the length of the language sample obtained to reduce the impact of bias contributed by transcript length. For example, one dialect density measure (DDM) is the ratio of utterances that contain one or more dialect-specific forms to the total number of utterances produced. Another DDM is the ratio of the total number of dialect-specific forms in the sample to the total number of words produced (Craig & Washington, 2000, 2002; Craig et al., 1998). Studies that have examined correlations between these DDMs and students' reading performance have found significant negative associations (Gatlin & Wanzek, 2015).

Both morphosyntactic and phonological AAE-specific forms have been quantified in research using measures of dialect density (Terry, 2006; Washington et al., 2018). AAE-specific morphosyntactic forms include variable past tense *-ed* marking (e.g., “He *walk* there yesterday,” where “yesterday” indicates that the action is past tense) and multiple negatives to intensify negation (e.g., “He *don't* see *nothin*”; Thompson et al., 2004; Washington & Craig, 2002). AAE-specific phonological forms include pronunciation of the printed “-ing” as /ɪn/ (e.g., “running” pronounced /rʌnɪn/) and initial “th-” as /d/ (e.g., “though” pronounced /doʊ/; Craig & Thompson et al., 2003; Thomas, 2007). These forms may appear both in the spoken and written language of AAE speakers (Ivy & Masterson, 2011; Patton-Terry & Connor, 2010). See Craig and Washington (2004) for a list of AAE-specific morphosyntactic and phonological forms.

African American individuals exhibit wide variability in AAE use, indicating first that not all African American students speak AAE and, secondarily, that dialect-specific forms may be produced differently in different contexts (Jencks, 1998; Terry et al., 2016; Thompson et al., 2004). Among students who do speak AAE, there exists a well-documented ability to dialect shift (i.e., adjust the frequency of use of AAE-specific forms) based on the context. For example, Horton-Ikard and Miller (2004) found that students produced AAE-specific forms at differing rates when asked to tell stories about past experiences compared to when they engaged in simple conversation with a Caucasian clinician. Similar results have been observed in comparisons of density of AAE-specific forms in sentence repetition tasks relative to storytelling, with GAE-based sentence repetition yielding lower density of AAE-specific forms than storytelling (Connor & Craig, 2006).

The ability to dialect shift fluently based on context and, in particular, to reduce use of AAE-specific forms in school-based contexts has been suggested to be an indicator of metalinguistic skill, which in turn may contribute to literacy development and broader academic achievement (Craig et al., 2009, 2014; Terry et al., 2012). To dialect shift fluently, an individual must not only develop awareness of dialect-specific and dialect-neutral forms, but also of contexts in which certain dialectal forms may be preferred and others stigmatized (Latimer-Hearn, 2020). Individuals generally are expected to develop these skills without explicit instruction or acknowledgement of biases underlying linguistic stigma (Terry et al., 2018), though there are exceptions (e.g., Edwards & Rosin, 2016; Johnson et al., 2017).

## AAE-Specific Forms in Writing

Writing may represent a context in which students are more explicitly taught to use fewer AAE-specific forms (Horton-Ikard & Pittman, 2010; Johnson et al., 2017). Overall, children who speak AAE tend to use fewer AAE-specific forms in written contexts compared to oral language (Craig et al., 2009; Patton-Terry & Connor, 2010), a trend that may be attributable to the formality of academic language more commonly expected in written communication relative to spoken communication (Charity Hudley et al., 2018; Puranik et al., 2019). High-achieving speakers of AAE seem to pick up on this trend, often “dialect shifting” or “code switching” without direct instruction, exhibiting fewer AAE-specific forms in their writing (Craig et al., 2009) and generally reducing the density of AAE-specific forms in their language as they get older (Ivy & Masterson, 2011). Highly stigmatized forms such as *ain't*, multiple negation, and habitual *be* are examples of some AAE-specific forms that appear less frequently in writing when compared to spoken communication (Ivy & Masterson, 2011).

Students who struggle academically, however, may not pick up on these implicit expectations (Craig et al., 2009; Hendricks & Diehm, 2020; Terry et al., 2010). Therefore, students with lower overall language skills and academic achievement may exhibit a higher density of AAE-specific forms in their writing.

An increasing number of studies have focused specifically on African American students' writing and the potential influence of AAE-specific forms on writing development. This work suggests that, among African American students who speak AAE, variability in spelling and written morphosyntax may be in part attributable to contrasts between spoken AAE and written academic language expectations (Gatlin & Wanzek, 2017; Patton-Terry & Connor, 2010; Puranik et al., 2019). For example, Horton-Ikard and Pittman (2010) examined the written language samples of 10th-grade African American students and observed some AAE-specific morphosyntactic forms in their writing. The density and diversity of these forms, however, were lower than that observed in their oral language (Horton-Ikard & Pittman, 2010). Ivy and Masterson (2011) compared written and oral language samples of African American students in third and eighth grade. They identified six grammatical forms specific to AAE for coding, including the zero forms of: verbal –*s*; plural –*s*; possessive –*s*; regular past tense –*ed*; *be* copula; and the *be* auxiliary. Among third graders, no statistically significant differences were found in the density of AAE-specific forms between oral and written modalities. However, eighth graders demonstrated a significantly higher density of AAE-specific forms in spoken language compared to written language, suggesting that older students had developed the ability to dialect shift within the written language context (Ivy & Masterson, 2011).

## This Study

Underlying, inherent language ability is not determined by mainstream versus nonmainstream dialect use and using a nonmainstream dialect in and of itself does not lead to literacy difficulties (Lee-James & Washington, 2018; Terry et al., 2018). Both children with and without language disorders may use dialect-specific forms with varying densities. Therefore, to examine the specific contribution of nonmainstream dialect density to reading achievement, accounting for both dialect-specific and dialect-neutral forms may be necessary (Oetting et al., 2016, 2019). In this study, we account for dialect-neutral language ability through two types of measures. First, we computed density measures for dialect-neutral variations from standardized written GAE (Fogel & Ehri, 2000), counting forms that would be considered ungrammatical in both AAE and standardized written GAE. For example, the sentence, “They run jump,” includes omission of the conjunction *and*. Conjunction omission violates the rules of both AAE and standardized written GAE, and therefore, would be considered a dialect-neutral variation. Second, we included measures of written language productivity (e.g., number of different words [NDW], total number of t-units [TNU]) to account for variance reduction due to sample length, which can lead to inaccurate estimations of language ability (Hendricks & Adlof, 2017).

Socioeconomic status, another key influencer of academic success (Dietrichson et al., 2017), may be confounded with the density at which individuals use dialect-specific forms (Charity et al., 2004). African American students are more likely to live in impoverished environments and attend underresourced schools due to systemic racism (Reardon et al., 2018, 2019). Consequently, to disentangle the precise relation between nonmainstream dialect density and achievement, socioeconomic status should be considered (Craig et al., 2009).

In this study, we sought to evaluate the predictive relation between African American students' written use of AAE-specific forms and their reading achievement. We included general writing productivity, family income, and dialect-neutral written variations as covariates. As noted above, it is well established that the three included covariates influence language, literacy, and overall academic performance. Therefore, their inclusion allows for a more precise examination of the unique relation between nonmainstream dialect-specific forms and literacy achievement. We examined written language samples produced by African American students between Grades 1–8 in response to a narrative prompt. We addressed the following questions:

1. What are the general descriptive characteristics of written, personal narrative language samples produced by African American students in Grades 1–8?
2. How often do AAE-specific morphosyntactic forms appear in the students' written language samples?
3. Does the density at which AAE-specific morphosyntactic forms appear in students' writing samples predict reading achievement when also accounting for dialect-neutral ungrammatical forms, writing productivity, and family income?

## Method

### Participants

This study utilized a subsample of school-age children who participated in the Florida Twin Project on Reading, Behavior, and Environment, a large study of twins attending schools throughout Florida (Taylor et al., 2019). Participants were selected from the larger study based on their caregivers' report of their race/ethnicity. All children whose parents reported their race/ethnicity as “African American” were included. This sample included 207 children, with 95 complete twin pairs and an additional 17 singleton participants whose twins did not complete the narrative prompt. Participants were in first through eighth grade, with an average age of 11.5 years. Demographic information for included children is provided in Table 1 at the child level.

**Table 1.** Participant demographics by child.

Characteristic	%	*n*	Characteristic	%	*n*
Child grade (*n* = 207)			Household income (*n* = 194)		
1st grade	7.7	16	Less than $10,000/yr	15.5	30
2nd grade	10.6	22	$10,000–29,000/yr	26.3	51
3rd grade	9.2	19	$30,000–49,000/yr	16.5	32
4th grade	9.7	20	$50,000–69,000/yr	16.5	32
5th grade	11.1	23	$70,000–89,000/yr	6.2	12
6th grade	15.0	31	More than $90,000/yr	19.1	37
7th grade	22.7	47	Child lunch status (*n* = 149)		
8th grade	14.0	29	Free or reduced	71.1	106
Child gender (*n* = 207)			No free/reduced	28.9	43
Female	51.2	106			
Male	48.8	101			

*Note.* yr = year.

### Procedure and Measures

Standardized, state-level achievement measures were administered by trained proctors as part of statewide testing required by normal school attendance. Test scores were uploaded into Florida's Progress Monitoring and Reporting Network. Schools also reported participants' eligibility for free or reduced-price lunch.

During the 2012–2013 school year, questionnaires and testing packets were mailed to the homes of participants enrolled in the larger project (Taylor et al., 2019). Parents completed self-report measures concerning their children's race and family socioeconomic status. Parent education was reported on a 8-point scale, with “1” indicating Grade 6 or less and “8” indicating graduate or professional school. Parents also reported yearly household income on a 12-point scale at $19,000 intervals. The value “1” specified an income below $10,000 and “12” indicated $210,000 or more per year.

Parents were also asked to administer a battery of achievement tests, including subtests from the Gates–MacGinitie Reading Test–Fourth Edition (GMRT-4; MacGinitie & MacGinitie, 2006) and the narrative writing prompt, to their twins using a scripted elicitation guide. Neither task requires special qualifications nor training, and both may be group administered. Written instructions included, “We would like for you to ‘play teacher’ and read the directions as if you were speaking to a classroom and monitor the twins so that you can ensure they do their work individually” (also see Daucourt et al., 2020). Parents were asked to report any testing that did not occur as instructed, and the investigators made case-by-case decisions on data quality for any inconsistencies described. All parents provided informed consent and children provided assent to participate as approved by the Florida State University Institutional Review Board.

#### Written Language Samples

Language samples provide snapshots of children's expressive language use and can reveal variations in language that predict later achievement (Horton-Ikard, 2010; Moyle et al., 2007; Rojas & Iglesias, 2013). Written narratives were elicited using the prompt, “One day when I got home from school…” in the child's natural setting (e.g., home) similar to written prompts used in other studies to examine school-age children's written language skills (Bahr et al., 2012; Dockrell et al., 2014). Children used pencils and were instructed to write on lined paper provided to the parents by the researchers. The writing task was untimed and not constrained in length; however, caregivers were told the activity should take 10–15 min. This elicitation strategy was selected because it is naturalistic, simple for caregivers to administer, and appropriate for a wide age range.

*Transcription of samples*. Undergraduate students enrolled in the speech-language pathology major typed the written samples into electronic transcript files. Transcripts were then formatted using Systematic Analysis of Language Transcripts (SALT) software and conventions (Miller & Iglesias, 2017), and segmented into t-units by undergraduate research assistants following conventions of segmenting writing samples (Price & Jackson, 2015). All research assistants received training in SALT transcription and segmentation and demonstrated at least 90% reliability with the first author on word-by-word transcription, t-unit segmentation, and SALT conventions on practice transcripts. Practice reliability was established on each of these components of transcription before research assistants began transcribing the research samples.

*Measures of written language productivity*. Several standard measures of language sampling were obtained from the formatted transcripts (Miller & Iglesias, 2017). Number of total words (NTW), a measure of transcript length and broad semantics, was obtained by counting the number of words written in the sample. The NDW, which is a measure of lexical diversity, was computed by summing the total number of different root words included in the sample. Mean length of t-unit, a measure analogous to mean length of utterance that quantifies morphosyntactic complexity, was computed by dividing the number of total morphemes in a sample by the total number of completed t-units in the sample. TNU, another measure of transcript length, was obtained by summing the number of t-units (Price & Jackson, 2015).

*Coding written language forms*. Two research assistants first independently coded each of the written narratives for grammatical, spelling, and punctuation forms that would be considered variations relative to standardized written GAE (Fogel & Ehri, 2000; Horton-Ikard & Pittman, 2010). The identified grammatical forms were then categorized as either (a) specific to African American English (S-AAE), or (b) dialect neutral forms that would be considered ungrammatical relative both to AAE and standardized written GAE (M-Neutral). A list of AAE-specific morphosyntax was used to categorize the forms (Craig & Washington, 2004; Washington & Craig, 2002). Variations from standardized written GAE that were consistent with AAE were identified as S-AAE. Those that were not consistent with AAE were identified as M-Neutral. For example, the sentence, “She walk to store earlier,” includes two grammatical variations relative to standardized written GAE: “She walk…earlier” includes a zero form past tense –*ed,* and “to store” includes an article omission. Zero form past tense –*ed* would be categorized as S-AAE because it is consistent with the morphosyntactic rules of AAE, whereas article omission would be categorized as M-Neutral because it is not consistent with the morphosyntactic rules of AAE or standardized written GAE.

We focused on grammar because African American students have been observed to incorporate more forms consistent with the morphosyntax of AAE than those consistent with the phonology of AAE in their writing (Thompson et al., 2004). The procedures used were aligned with prior work examining general writing variations conducted by Scott and Windsor (2000) and work examining AAE-specific forms in writing conducted by Horton-Ikard and Pittman (2010).

Both the research assistants who coded the written samples completed transcription and coding training protocols directed by the first author. Training included (a) explicit instruction of definitions and contextual examples of AAE-specific forms (Craig & Washington, 2004), (b) guided practice on 10 written samples, (c) independent practice on 15 samples with specific feedback and line-by-line fidelity scoring provided for each sample, and (d) reliability testing requiring 90% coding fidelity for individual forms on 10 samples.

*Density measures*. Token-based measures of density were calculated based on the written samples both for S-AAE and for M-Neutral (Oetting & McDonald, 2002). The measures were based on the three DDMs (density by total words, density by t-units, and density by t-units containing a target form) that have been used to examine dialect use in written samples and that are considered more robust measures than raw number of form occurrences (Ivy & Masterson, 2011; Schachter & Craig, 2013).

For the AAE-specific forms (S-AAE), density by total words (DDM_w_) was obtained by summing the total forms identified as S-AAE in the analysis set, and then dividing by the words in the sample. Density by t-units (DDM_t_) was calculated by again summing the total number of S-AAE forms, but then dividing by the number of t-units in the sample. Finally, density by t-units containing a target form (DDM_d_) was computed by counting the number of t-units in each sample that included one or more S-AAE form and dividing by the TNU in the sample.

For the dialect-neutral ungrammatical forms (M-Neutral), three values were also computed to provide comparable density measures. Density by total words (Neutral_w_) was computed by summing the M-Neutral forms identified in the sample, and then dividing by the total words in the sample. Density by t-units (Neutral_t_) was calculated by again summing the M-Neutral forms identified, and then dividing by the number of t-units in the sample. Neutral_d_ was computed summing the number of t-units in each sample that included one or more M-Neutral form and then dividing by the number of total t-units in the sample.

*Reliability*. Reliability for the written samples was established between the two trained research assistants. Research assistants double coded all 207 of the transcripts. Reliabilities were computed by dividing the total agreements by the sum of agreements plus disagreements. Coding reliability was good, measured at 97.4% for S-AAE, 95.9% for M-Neutral, 99.6% for t-unit segmentation, and 99.5% for morpheme segmentation. Discrepancies were resolved by the first author after obtaining reliability values.

#### Outcome Measures

Three measures of language and literacy were included to profile reading achievement for participating students. These included a state-level high-stakes test (Florida's Comprehensive Assessment Test 2.0 [FCAT]), a norm-referenced assessment designed for progress monitoring (Florida Assessments and Instruction in Reading [FAIR]), and a reading vocabulary assessment administered at home (GMRT-4). These three measures were selected to represent a range of skills important to longitudinal academic achievement.

FCAT (Florida Department of Education, 2013) is a state-wide high-stakes assessment administered annually near the end of the academic year. The FCAT is designed for Grades 3–12 and covers content areas including reading, writing, math, and science. Students' developmental scaled scores from the FCAT reading assessment were used in this study. Internal consistency reliability (Cronbach's alpha) is reported to be .88–.92 for FCAT Reading.

The FAIR (Foorman et al., 2009) was designed to assess students' global literacy skills as a progress-monitoring indicator and predictor of FCAT Reading performance. Administered in the fall, winter, and spring of the academic year, the FAIR is a norm-referenced computer-adaptive screening and diagnostic measure. It is aligned with state language arts standards and scaled for grades K–12. The FAIR reading comprehension, maze (reading fluency), and word analysis (spelling) subtests were administered to all students across Grades 3–7 in 2012–2013, and therefore, were selected for the present analyses. Standard scores from all three testing occasions of the school year were included. Internal consistency reliability ranged from .86 to .92 for the included subtests.

Subtests of the GMRT-4 (MacGinitie & MacGinitie, 2006) were also administered by participants' caregivers following a scripted elicitation guide sent via mail (see Daucourt et al., 2020). The GMRT-4 is a paper–pencil test designed for individuals in kindergarten through adulthood. The full assessment is often employed as a diagnostic tool to provide information about students' strengths and weaknesses in reading. Different subtests are administered for reading vocabulary by grade level. Students in Grades 1–2 complete the Word Decoding subtest, for which the student views a picture (e.g., practice item: pig), and a list of four orthographically similar written words (e.g., *big, fig, pig, dig*). The child is directed to select the word that corresponds with the picture. Students in Grades 3 and up complete the Vocabulary subtest, for which the student views a sentence with a word underlined (e.g., practice item: *She felt happy*), and a list of four written words (e.g., *sleepy, hot, ready, glad*). The student is directed to select the word that is a synonym or definition of the underlined word. The test manual reports construct validity estimates of .79 to .81; test–retest reliability between .85 and .90; and internal reliability of .96 (MacGinitie & MacGinitie, 2006).

### Analyses

To address the first research question, data were first examined descriptively to provide an overall picture of students' writing. Descriptive data were obtained across the entire sample for background characteristics, standard measures of language sampling, and measures of academic achievement. In response to the second research question, samples were evaluated for frequency of occurrence of each AAE-specific form (S-AAE).

To evaluate the relation between written density of AAE-specific forms and academic achievement as indicated by the third research question, structural equation modeling (SEM) was used. Data normality and multivariate linearity were evaluated through the psych (Revelle, 2019) and ggplot2 (Wickham, 2016) packages in R (R Core Team, 2020). Age was regressed out of the standard measures of language sampling, S-AAE, M-Neutral, and GMRT-4 and FCAT scores to obtain values comparable across all ages included in the sample (FAIR data already accounted for age). Values were then *z* scored to provide a consistent metric for interpretation across the model. To address the nesting of twins within families, twin pairs were initially randomly divided into two samples and examined for substantive differences. As no differences were observed between the groups, the sample was recombined and family nesting was accounted for in subsequent modeling. This decision was made based on the body of work that suggests that research findings from twin samples are generalizable to broader populations (e.g., Christensen et al., 2006; Walker et al., 2004).

Next, confirmatory factor models were evaluated for each of the latent constructs of interest using Mplus 8.4 (Muthén & Muthén, 2019). A latent construct of writing productivity was measured through NTW, TNU, and NDW. M-Neutral density was constructed from the three M-Neutral density measures (Neutral_w_, Neutral_t_, Neutral_d_). Similarity, S-AAE density included the DDMs (DDM_w_, DDM_w_, DDM_d_). Finally, the multiple measures available from the FAIR (i.e., three time points for word analysis, mazes, and reading comprehension) were examined as contributors to a single latent factor. Each structure was examined individually to confirm goodness of fit before being included in the larger model.

After the latent factor structures were established, the hypothesized structural model was analyzed in Mplus 8.4 (Muthén & Muthén, 2019). Model fit was assessed following descriptions by Kline (2011) and Chen et al. (2008). Generally, a root-mean-square error of approximation (RMSEA) below .10, a comparative fit index (CFI) and Tucker Lewis index (TLI) above .90, and the standardized root-mean-square residual (SRMR) below .08, were considered indicators of reasonable global fit, although values were evaluated collectively and with preference for more stringent values. Individual parameter estimates were also examined for evidence of misfit (e.g., negative residual variance; Kline, 2011).

## Results

Descriptives for background characteristics, standard measures of language sampling, and reading achievement scores are provided in Table 2. Within the present participant sample, 150 students included at least one instance of an S-AAE form in their written narratives. All but seven of the participants had at least one instance of an M-Neutral form in their writing. Descriptive information for the language sampling measures is provided by grade in Table 3. Descriptive information for all measures is disaggregated by presence of S-AAE forms in Supplemental Material S1. 

**Table 2.** Sample descriptives.

Characteristic		Full sample				
		*n*	*M*	*SD*	Min	Max
S-AAE forms		207	2.38	3.31	0	22
M-Neutral forms		207	10.85	9.25	0	55
Age (years)		207	11.49	2.39	6.75	15.33
Language sample microstructure measures						
TNU		207	17.41	13.52	1	104
MLTU (morphemes)		207	9.35	2.99	4.2	30
NDW		207	75.57	43.78	10	267
NTW		207	144.37	104.23	12	753
Achievement measures						
GMRT-4		199	517.71	51.04	350	653
FCAT		104	222.13	23.46	153	272
FAIR1	Reading comprehension	95	94.52	12.36	72	131
	Maze	94	94.26	13.46	71	131
	Word analysis	93	96.63	14.77	60	127
FAIR2	Reading comprehension	92	95.89	12.90	69	144
	Maze	89	99.83	14.86	74	140
	Word analysis	88	95.57	15.60	63	133
FAIR3	Reading comprehension	87	100.05	14.49	73	155
	Maze	87	101.56	15.36	77	140
	Word analysis	84	96.40	14.88	60	138

*Note.* Min = minimum; Max = maximum; S-AAE = African American English–specific morphosyntactic forms; M-Neutral = dialect neutral forms; TNU = total number of t-units; MLTU = mean length of t-unit in morphemes; NDW = number of different words; NTW = number of total words; GMRT-4 = Gates–MacGinitie Reading Tests (reading vocabulary), 4th Edition; FCAT = Florida's Comprehensive Assessment Test; FAIR1 = Florida Assessments for Instruction in Reading Fall 2012; FAIR2 = FAIR Winter 2012; FAIR3 = FAIR Spring 2013; Maze = reading fluency.

**Table 3.** Writing sample descriptive statistics by grade.

Grade	*n*	S-AAE		M-Neutral		TNU		MLTU-m		NDW		NTW	
		*M*	*SD*	*M*	*SD*	*M*	*SD*	*M*	*SD*	*M*	*SD*	*M*	*SD*
1	16	1.62	1.89	9.38	5.43	8.12	3.16	7.13	2.51	31.94	12.28	53.00	25.64
2	22	1.55	2.26	11.27	10.48	13.36	10.08	7.59	1.58	50.41	34.37	98.36	80.80
3	19	1.89	1.66	8.89	7.29	10.63	5.04	8.63	1.77	47.63	17.76	86.21	44.25
4	20	2.70	4.61	11.55	8.65	19.95	9.82	8.69	2.40	80.20	27.30	153.55	67.28
5	23	2.35	1.85	11.13	6.66	20.43	11.33	9.37	2.04	87.96	36.41	174.04	91.60
6	31	2.68	3.53	11.52	10.67	21.23	16.68	10.04	4.42	87.87	47.75	169.97	117.41
7	47	3.40	4.71	12.47	11.77	19.47	16.32	10.00	2.93	87.36	44.08	169.49	121.49
8	29	1.55	1.57	8.59	6.92	18.48	14.69	11.00	2.50	91.72	53.12	169.86	112.99

*Note.* S-AAE = African American English–specific morphosyntactic forms; M-Neutral = dialect neutral forms; TNU = total number of t-units; MLTU = mean length of t-units in morphemes; NDW = number of different words; NTW = number of total words.

**Figure 1.**
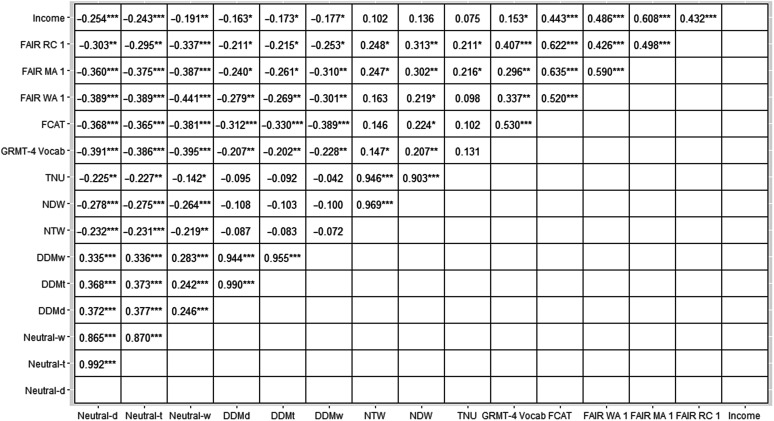
Correlations among z scored variables. *p < .05, **p < .01, ***p < .001. FCAT = Florida's Comprehensive Assessment Test (Reading); GMRT-4 = Gates–MacGinitie Reading Tests–Fourth Edition; RC1 = Reading Comprehension on Florida Assessment for Instruction in Reading (FAIR) in Fall 2012; M1 = Maze (reading fluency) on FAIR in Fall 2012; WA1 = word analysis (spelling) on FAIR in Fall 2012; TNU = total number of t-units; MLTU = mean length of t-unit; NDW = number of different words; NTW = number of total words; DDM values are density measures for AAE-specific morphosyntactic forms (S-AAE); Neutral values are density measures for dialect-neutral forms (M-Neutral).

Sample length and lexical diversity was associated with age. Older students produced longer samples with more words: TNU *r* = .23, *p* = .005; NTW *r* = .32, *p* < .001; NDW *r* = .39, *p* < .001. Students' ages were negatively associated with DDM_w_ (*r* = −.21, *p* = .009), but not with DDM_t_ or DDM_d_: *r* = −.06, *p* = .494, and *r* = −.04, *p* = .640, respectively. Student age also was negatively associated with all measures of M-Neutral density: Neutral_w_ *r* = −.37, *p* < .001; Neutral_t_ *r* = −.22, *p* = .001; Neutral_d_ *r* = −.22, *p* = .002. Correlations among z scored variables are presented in Figure 1.

Unsurprisingly, given the past tense formulation of the narrative prompt, zero form past tense –*ed* appeared the most frequently (*M* = 1.07 per sample, *SD* = 1.75), followed by zero form plural –*s* (*M* = 0.43 per sample, *SD* = 1.05), then subject–verb shifts (*M* = 0.34 per sample, *SD* = 0.85). Six AAE-specific morphosyntactic forms were not produced in any of the samples. These included remote past *been,* regularized reflexive pronouns, invariant *be,* double copula/auxiliary/modal, use of *ain't,* and completive *done*. Average occurrences of each coded form is provided in Table 4 (AAE-specific) and Table 5 (M-Neutral).

**Table 4.** AAE-specific morphosyntactic forms appearing in the written samples.

Form	Occurrences per sample *M* (*SD*)	Form	Occurrences per sample *M* (*SD*)
Zero form past tense –*ed*	1.07 (1.75)	Zero form present progressive –*ing*	0.04 (0.23)
Zero form plural –*s*	0.43 (1.05)	Double marking	0.03 (0.20)
Subject-verb shifts	0.34 (0.85)	Existential *it*	0.03 (0.20)
Preterite *had*	0.29 (0.64)	Multiple negation	0.03 (0.16)
Zero form articles	0.27 (0.59)	Undifferentiated pronoun case	0.01 (0.12)
Zero form copula	0.21 (0.65)	Fitna/sposeta/bouta	0.01 (0.08)
Zero form possessive –*s*	0.19 (0.65)	Remote past *been*	0
Zero form infinitive *to*	0.11 (0.33)	Regularized reflexive pronoun	0
Indefinite article *a*	0.07 (0.33)	Invariant *be*	0
Zero form prepositions	0.06 (0.24)	Double copula/auxiliary/modal	0
Zero form auxiliary	0.05 (0.23)	Use of *ain't*	0
Appositive pronouns	0.05 (0.24)	Completive *done*	0

*Note.* AAE = African American English.

**Table 5.** M-Neutral morphosyntactic forms coded in the written samples.

Form	Example(s)	Occurrences per sample		
		*M*	*SD*	Range
Homophone substitution	She took **there | their** books away.	2.67	2.73	0–17
Nonseriation run-on sentences	(2+ clauses not connected with a conjunction)	1.88	2.19	0–14
Whole-word omission	My brothers __ against each other.	1.24	2.00	0–14
Tense change (not contextually indicated)	Everyone **says** hello, then she **gave** me a gift.	0.70	1.21	0–6
Seriation “then…and then…”	(3+ independent clauses in a row beginning with a conjunction)	0.52	0.98	0–4
Whole-word addition	The wings were **the** tipped with brown.	0.46	0.85	0–4
Regularization of past tense –*ed* ^a^	I **lefted** my keys there.	0.03	0.19	0–2
Other grammatical variations	I wish cats **can** fly.	3.36	4.28	0–23
	I saw **she** yesterday.			
	**An** geese chase the cat.			

a Although not included in all lists of grammatical forms specific to AAE, regularization of past tense –*ed* may be considered an AAE-specific form (e.g., Pruitt & Oetting, 2009; Wolfram, 2004). We conducted sensitivity analyses with and without –*ed* regularization included in the statistical models. Findings did not differ based on the classification of this specific form.

### Factor Analyses

**Figure 2.**
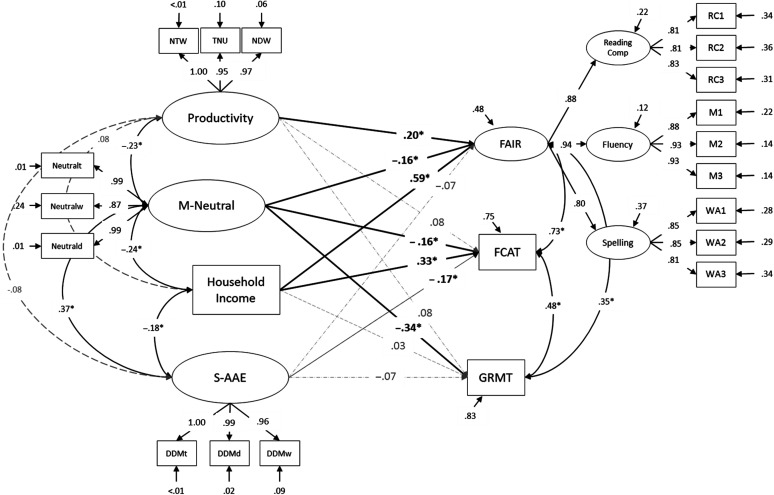
Full model including all outcome measures with standardized path coefficients. Solid lines indicate significant paths (*p* < .05). NTW = number of total words; TNU = total number of t-units; NDW = number of different words; DDM values are density measures for AAE-specific morphosyntactic forms (S-AAE); NM values are density measures for dialect-neutral forms (M-Neutral); RC = reading comprehension; Maze = reading fluency; WA = word analysis; 1 = score in Fall 2012; 2 = score in Winter 2013; 3 = score in Spring 2013; FCAT = Florida's Comprehensive Assessment Test (Reading); GMRT = Gates–MacGinitie Reading Tests–Fourth Edition.

Confirmatory factor models constructed for each of the latent constructs of interest all fit the data well, with global fit statistics well within preferred ranges (Kline, 2011) and no evidence of misfit observed in the parameter estimates or residuals. The FAIR factor was best represented by a three-dimensional structure with a second-order latent factor for overall literacy (see “FAIR” in Figure 2). The standardized factor loadings obtained for these in the structural model framework are available in Supplemental Material S2.

### SEM

The hypothesized structural model fit the data reasonably, with global fit statistics within the preferred ranges. The RMSEA was 0.069 (90% CI [0.058,0.080]). The model yielded a CFI of 0.957, a TLI of 0.946, and an SRMR of 0.068. The model accounted for 25.5% of the variance in students' FCAT scores, 16.9% of the variance in GMRT scores, and 51.5% of the variance in FAIR. See Figure 2.

The density at which S-AAE forms appeared in the writing samples did not significantly predict FAIR or GMRT scores above and beyond the other predictors: FAIR (−0.07, *SE* = 0.10, *p* = .522) and GMRT (−0.07, *SE* = 0.07, *p* = .294). S-AAE did meet *p* < .05 criteria for significantly predicting students' FCAT scores (−0.17, *SE* = 0.10, *p* = .038). Conversely, the density at which dialect-neutral forms (M-Neutral) appeared did predict all three outcome measures significantly: FAIR (−0.16, *SE* = 0.08, *p* = .033), FCAT (−0.16, *SE* = 0.07, *p* = .024), and GMRT (−0.34, *SE* = 0.08, *p* < .001). Narrative productivity only significantly contributed to predicting FAIR (0.20, *SE* = 0.09, *p* = .021). Household income did not significantly predict GMRT scores, but it did predict FAIR (0.59, *SE* = 0.09, *p* < .001) and FCAT (0.34, *SE* = 0.08, *p* < .001).

To assess the robustness of the results, the hypothesized model was compared against one nested model using Satorra–Bentler chi-square difference testing with a correction factor for MLR (Satorra & Bentler, 2010). The comparison model included constraints for all of the path coefficients between S-AAE and the outcome measures set to zero (see Supplemental Material S3). Global model fit for the nested model was good: RMSEA = 0.069 (90% CI [0.058,0.079]), CFI = 0.956, TLI = 0.947, SRMR = 0.073. The constrained model was not a significantly worse fit to the data, χ^2^(3) = 4.08 with 0.96 correction, *p* = .253, suggesting S-AAE was not a key predictor of students' test scores after accounting for the other values in the model.

Generally, .80 power is acceptable to be confident in nonsignificant results for the overall model in SEM. For a model with over 100 degrees of freedom, such as the present model, a minimum of 178 participants are needed for the test of not-close fit, which is desired when the RMSEA is greater than 0.05 (MacCallum et al., 1996). This study had approximately 0.87 power, given the sample size was greater than 200 and the degrees of freedom were above 100, indicated that the model was adequately powered.

## Discussion

The primary purpose of this article was to examine the predictive relation between African American students' density of use of S-AAE morphosyntactic forms in writing and their reading achievement. We sought to evaluate this relation in the context of including income, writing productivity, and density of dialect-neutral ungrammatical forms as covariates. We examined measures of literacy and reading vocabulary as outcomes. This work was conducted to contribute to researchers' and practitioners' understanding of nonmainstream dialect use in academic contexts to inform the continued development of increasingly effective approaches for supporting the education of African American students.

In the context of these written samples, we found that the density at which S-AAE forms appeared did not substantially contribute to predicting students' performance on the measures of academic achievement when accounting for household income, writing productivity, and dialect-neutral forms. Rather, the density of forms considered to be ungrammatical both in AAE and standardized written GAE (i.e., M-Neutral) emerged as the most consistent significant predictor of students' scores on standardized achievement measures. This central finding that dialect-neutral ungrammatical forms, rather than AAE-specific forms, predicted test performance underscores the importance of general language skills in students' overall reading, writing, and academic development. Critically, the ability to acquire language skills is not contingent on use of any particular dialect (Lee-James & Washington, 2018; Terry et al., 2018). Regardless of spoken dialect, students demonstrate varying levels of underlying language ability (Johnson & Gatlin-Nash, 2020).

In the present work, findings suggest that written language productivity (Price & Jackson, 2015) and dialect-neutral ungrammatical forms are reflective of general underlying language ability. Production of longer written narratives suggests more fluent and less effortful writing, which in turn reflects stronger underlying expressive language skills. Similarly, use of few or no forms that are ungrammatical both in AAE and standardized written GAE indicates strong morphosyntactic knowledge, which also reflects higher underlying language ability. These dialect-neutral measures emerged as the key drivers of students' achievement, with the density of AAE-specific morphosyntactic forms serving as correlates of, but not unique contributors to, literacy performance. This may indicate that a reduced focus on AAE-specific form variation in students' writing is warranted. Rather, pending further empirical study, practitioners and researchers may find that African American students' writing development may be more effectively supported through an emphasis on dialect-neutral forms that explicitly teach children more nuanced rules, such as verb agreement in complex sentences, to encourage general language development (Johnson & Gatlin-Nash, 2020).

### Secondary Findings

Descriptively, the students produced highly variable written samples in terms of length (i.e., TNU and NTW) and lexical diversity (NDW). Some of this variability may be attributable to the wide age range of the sample, as age was positively correlated with the language sampling measures. However, the samples were diverse not only in length and lexical diversity, but also in the rate at which AAE-specific and dialect-neutral forms occurred.

Examination of the rate at which AAE-specific (S-AAE) forms appeared in the written samples revealed that the students in this study used overall lower rates of S-AAE compared to those observed in studies of spoken language (e.g., Gatlin & Wanzek, 2015). This finding is aligned with previous work indicating that African American individuals use S-AAE at variable rates in different contexts (Charity et al., 2004), and that students typically use fewer AAE-specific forms in writing compared to oral language (Ivy & Masterson, 2011). We also observed that density of S-AAE forms was negatively associated with income (all DDMs, see Figure 1) and with age (at least for DDM_w_). This finding is consistent with prior work indicating that students from higher income homes generally use S-AAE forms at lower densities than students from lower income homes (Charity et al., 2004) and that older students tend to incorporate fewer S-AAE forms in their writing compared to younger students (Ivy & Masterson, 2011).

Several S-AAE forms commonly observed in spoken language (e.g., use of *ain't* and invariant *be*) were absent from the participants' written responses in this study. This potential discrepancy between samples of spoken and written language corroborates previous findings regarding context-dependency of S-AAE forms. Specifically, previous research purports that students use AAE-specific forms differentially in their writing compared to their spoken language (Cronnell, 1984; Ivy & Masterson, 2011). The AAE-specific forms observed to occur at high rates, such as zero form past tense –*ed,* reflected the past tense context of the prompt.

In considering prediction of academic achievement, the finding that household income predicted achievement scores is consistent with a large body of literature suggesting children from low socioeconomic backgrounds are at risk for disproportionately low academic performance (Dietrichson et al., 2017) due to structural and systemic biases. Furthermore, the finding that language sample length and lexical diversity significantly predicted literacy scores is consistent with research indicating that narrative language is important to reading development (Gardner-Neblett & Iruka, 2015).

### Limitations

To consider the results from the present work as accurately as possible, it is essential to recognize that the complexity of language (and language variation) cannot be fully captured by coding approaches such as those used in the present work. We aimed to build upon prior work that has examined dialect-specific grammatical forms in students' expressive language, and to highlight the fact that both dialect-specific and dialect-neutral language ability have not often been accounted for in models of reading development. Token-based coding by dialect is not an easy task, however, because of the wide variation in individual dialectal exposure and use. Furthermore, we cannot know the exact reason for any given grammatical error or linguistic variation observed in students' writing samples. In this study, for example, it is possible that some forms that were coded as “dialect-neutral” were in fact consistent with individual students' language experiences. It is simultaneously possible that some forms that were coded as “AAE-specific” were not consistent with students' specific dialectal backgrounds.

Contextually, the present findings were obtained from a sample of African American student participants who lived in the southern region of the United States, specifically in Florida. We operationalized AAE-specific morphosyntactic forms based on prior work (Thompson et al., 2004; Washington & Craig, 2002), but no dialect is uniform across all speakers (e.g., Berry & Oetting, 2017). Variable external factors can influence individual use of specific linguistic forms, within and outside those specified in this study. Future work would benefit from more precise consideration of both within-dialect linguistic variation and written variation consistent with AAE-specific phonological forms (Kohler et al., 2007; Patton-Terry & Connor, 2010). We also recommend specific examination of dialect-neutral forms, based in a priori identification of dialect-neutral forms that are ungrammatical. In the present work, we used a reverse-identification approach, first identifying all instances of variations from standardized written GAE and then categorizing forms based on their consistency with AAE. This may explain the lower reliability observed for M-Neutral coding (95.9%) compared to S-AAE coding (97.4%). Coders may have been more likely to categorize forms they were uncertain of as “other grammatical variations” (a category only available for M-Neutral).

It is important to interpret the findings considering the statistical limitations of this work. First, an inherent limitation of SEM is the inability to statistically compare equivalent models (Kline, 2011). A different structure of the same variables (e.g., a mediation model) would produce an equivalent fit to the data. Although additional structures were considered, at present, we believe that the tested model represented the closest fit with current theory. However, we recognize that, as theory develops over time, alternative structures may more accurately represents the relations among the included variables.

Missing data also may have influenced the results. It is reasonable to infer that the primary results are relatively stable, given that the central finding replicated across all three outcome measures. However, missing data particularly in students' FCAT, as a single indicator in the model, reduced the statistical power to discern which predictors contributed significantly to FCAT (high-stakes reading achievement) performance. Missing data for FAIR (progress monitoring for reading) may be less concerning given the latent factor structure, which grants some robustness to missing data.

The results are also limited by the available assessment data, including some measures that were administered by parents (such as the GMRT-4). It should be noted that the available measures assessed selected aspects that are among a constellation of skills purported to contribute to literacy and academic achievement. It would be interesting in future studies to also consider the relation between other performance measures (such as morphological knowledge). Furthermore, it should be noted that a multitude of unmeasured factors (e.g., approach to writing instruction, teacher–student dialect match) may have also contributed to variability or served a moderating role.

Finally, we encourage continued examination of dialect-neutral forms across nonmainstream dialects. Although not within the scope of this article, specification of which linguistic forms maximally predict students' long-term language and reading abilities may be a key area for future work. Different forms may be important to emphasize within different instructional modalities (i.e., spoken vs. written). This remains an area of need in the literature to focus on maximizing educational outcomes for students from all backgrounds. A holistic approach to the assessment of students' writing, considering both broad dialect-neutral skills and dialect-specific forms together, may be essential to predicting students' academic outcomes (Johnson & Gatlin-Nash, 2020; Puranik et al., 2019).

### Implications for Practice

Our findings indicate that AAE, in and of itself, is not a barrier to written language development. Rather, general underlying language ability, as evidenced by the frequency of occurrence of dialect-neutral ungrammatical forms, is a key indicator of how students will perform on tasks such as those measured in this study. Therefore, it is important for speech-language pathologists and educators to leverage what we do know about dialectal variation to support speakers of AAE. This work must be conducted using culturally sustaining pedagogies that acknowledge the social context of systemic racism, both to support individual students' identities and to reduce linguistic stigmatization (Baker-Bell et al., 2017; Wynter-Hoyte et al., 2019).

The relationship between general language skills, reading, and writing ability has been well documented over time (Language and Reading Research Consortium, 2015). While determining whether a student has a language difference or a delay may be challenging, it is important to keep in mind that overlaps can and do exist in students who are nonmainstream dialect speakers, those who have language delays, those from disadvantaged backgrounds, and those who have true language disorders. Laing and Kamhi (2003) suggest our assessment practices for students from culturally and linguistically marginalized populations should involve alternate measures, such as dynamic assessment, language sampling (as used in this study), and processing-based tasks (e.g., working memory, nonword repetition; Stockman, 2010). Measures such as these provide a wealth of knowledge above and beyond what can be gleaned from standardized assessments alone. From this assessment information, practitioners can then intervene by explicitly supporting students' meta-awareness of morphosyntax through incorporating contrasts of AAE-specific and neutral forms in different contexts, such as writing. This will help AAE speakers who also have language deficits to increase their foundational language skills (Johnson & Gatlin-Nash, 2020).

## Conclusions

Although several of the background variables included in this work did significantly predict students' reading achievement, a substantial amount of variance in scores remained unexplained in the final model. Less than 30% of the variance in reading vocabulary and high-stakes reading scores and approximately 50% of the variance in FAIR reading was accounted for in the structural model. These results support the ongoing need to better understand the language, literacy, and overall academic development of students from all backgrounds. As practitioners focus explicitly on strengthening students' general underlying language skills through an emphasis on high-level, complex grammatical concepts (such as the dialect-neutral forms mentioned in this study) and evidence-based reading instruction, we hope to see a rise in the reading performance of African American students.

## Supplementary Material

10.1044/2021_AJSLP-20-00263SMS1 Supplemental Material S1 Sample descriptives by group.

10.1044/2021_AJSLP-20-00263SMS2 Supplemental Material S2 Standardized factor loadings from factor models.

10.1044/2021_AJSLP-20-00263SMS3 Supplemental Material S3 Comparison model including constraints for all of the path coefficients between S-AAE and the outcome measures set to zero.
