# Supplementary material for: Language Variation in the Writing of African American Students: Factors Predicting Reading Achievement
Source: Am J Speech Lang Pathol. 2021 Nov 1;30(6):2653–67. doi: 10.1044/2021_AJSLP-20-00263 (PMC9132061; doi:10.1044/2021_AJSLP-20-00263)
Supplement: Supplemental Material S1 [file AJSLP-30-2653-s001.pdf]

Supplemental Material S1. Sample descriptives by group.

Characteristic	Students with at least one S-AAE Form in Writing			Students with no S-AAE Forms in Writing			Full Sample		
	n	*M* (*SD*)	*Min–Max*	*n*	*M* (*SD*)	*Min–Max*	*n*	*M* (*SD*)	*Min–Max*
M-Neutral Forms	150	12.44 (9.91)	0–55	57	6.67 (5.42)	0–27	207	10.85 (9.25)	0–55
Age (years)	150	11.65 (2.30)	6.75–15.00	57	11.10 (2.58)	6.75–15.33	207	11.49 (2.39)	6.75–15.33
Income[1]	139	3.53 (2.16)	1–9	55	3.40 (1.94)	1–8	194	3.49 (2.09)	1–9
Language Sampling Measures									
TNU	150	19.61 (14.60)	1–104	57	11.63 (7.64)	1–36	207	17.41 (13.52)	1–104
MLTU (morphemes)	150	9.27 (2.54)	4.33–22	57	9.54 (3.96)	4.2–30	207	9.35 (2.99)	4.2–30
NDW	150	83.51 (45.60)	10–267	57	54.67 (30.09)	11–120	207	75.57 (43.78)	10–267
NTW	150	162.39 (111.17)	13–753	57	96.96 (62.66)	12–273	207	144.37 (104.23)	12–753
Achievement Measures									
GMRT-4	143	517.27 (50.96)	350–653	56	518.84 (51.68)	387–653	199	517.71 (51.04)	350–653
FCAT	81	221.38 (24.42)	153–272	23	224.74 (19.94)	177–252	104	222.13 (23.46)	153–272
FAIR1 Reading Comp	73	93.92 (12.83)	72–131	22	96.50 (10.67)	78–117	95	94.52 (12.36)	72–131
FAIR1 Maze	72	94.28 (13.72)	71–131	22	94.18 (12.89)	71–122	94	94.26 (13.46)	71–131
FAIR1 Word Analysis	72	96.69 (15.86)	60–127	21	96.43 (10.51)	81–118	93	96.63 (14.77)	60–127
FAIR2 Reading Comp	70	96.33 (13.24)	69–144	22	94.50 (11.95)	72–112	92	95.89 (12.90)	69–144
FAIR2 Maze	68	99.72 (15.28)	74–140	21	100.19 (13.78)	77–131	89	99.83 (14.86)	74–140
FAIR2 Word Analysis	68	95.82 (15.81)	63–133	20	94.70 (15.25)	66–129	88	95.57 (15.60)	63–133
FAIR3 Reading Comp	66	99.86 (15.67)	73–155	21	100.62 (10.21)	82–116	87	100.05 (14.49)	73–155
FAIR3 Maze	66	101.53 (15.46)	77–140	21	101.67 (15.43)	81–131	87	101.56 (15.36)	77–140
FAIR3 Word Analysis	64	97.36 (15.92)	60–138	20	93.35 (10.67)	76–114	84	96.40 (14.88)	60–138

[1]On a 1-12 scale, with 1 = *less than $10,000/year* and each 1-unit increase representing $19,000. S-AAE = African American English-specific morphosyntactic forms; M-Neutral = dialect neutral forms relative to standard written English; TNU = Total number t-units; MLTU = mean length of t-unit in morphemes; NDW = number different words; NTW = number total words; GMRT = Gates-MacGinitie Reading Tests (reading vocabulary), 4th Edition; FCAT = Florida's Comprehensive Assessment Test; FAIR1 = Florida Assessments for Instruction in Reading fall 2012; FAIR2 = FAIR winter 2012; FAIR3 = FAIR spring 2013; RC = reading comprehension; Maze = reading fluency; WA = word analysis.
