# Supplementary material for: Language Variation in the Writing of African American Students: Factors Predicting Reading Achievement
Source: Am J Speech Lang Pathol. 2021 Nov 1;30(6):2653–67. doi: 10.1044/2021_AJSLP-20-00263 (PMC9132061; doi:10.1044/2021_AJSLP-20-00263)
Supplement: Supplemental Material S2 [file AJSLP-30-2653-s002.pdf]

Supplemental Material S2. Standardized factor loadings from factor models.

Factor	Variables	Factor Loadings	*SE*	*p*	R^2
AAE-Specific Forms (S-AAE)	DDMt	1.00	—	—	1.00
	DDMd	.99	.01	< .001	.98
	DDMw	.96	.01	< .001	.91
Dialect Neutral Forms (M-Neutral)	$Neutral_t$	1.00	.01	—	1.00
	$Neutral_w$	.87	.02	< .001	.76
	$Neutral_d$	.99	.01	< .001	.99
Writing Productivity	NTW	1.00	—	—	1.00
	TNU	.95	.02	< .001	.90
	NDW	.97	.01	< .001	.94
Spelling	WA1	.85	.05	< .001	.72
	WA2	.85	.04	< .001	.72
	WA3	.81	.06	< .001	.66
Fluency	M1	.88	.03	< .001	.78
	M2	.93	.02	< .001	.86
	M3	.93	.02	< .001	.86
Reading Comprehension	RC1	.81	.05	< .001	.66
	RC2	.81	.04	< .001	.65
	RC3	.83	.04	< .001	.69
FAIR	Fluency	.94	.04	< .001	.87
	Spelling	.80	.06	< .001	.63
	Reading	.88	.05	< .001	.78
