# Supplementary figures and images for: Language Variation in the Writing of African American Students: Factors Predicting Reading Achievement
Source: Am J Speech Lang Pathol. 2021 Nov 1;30(6):2653–67. doi: 10.1044/2021_AJSLP-20-00263 (PMC9132061; doi:10.1044/2021_AJSLP-20-00263)

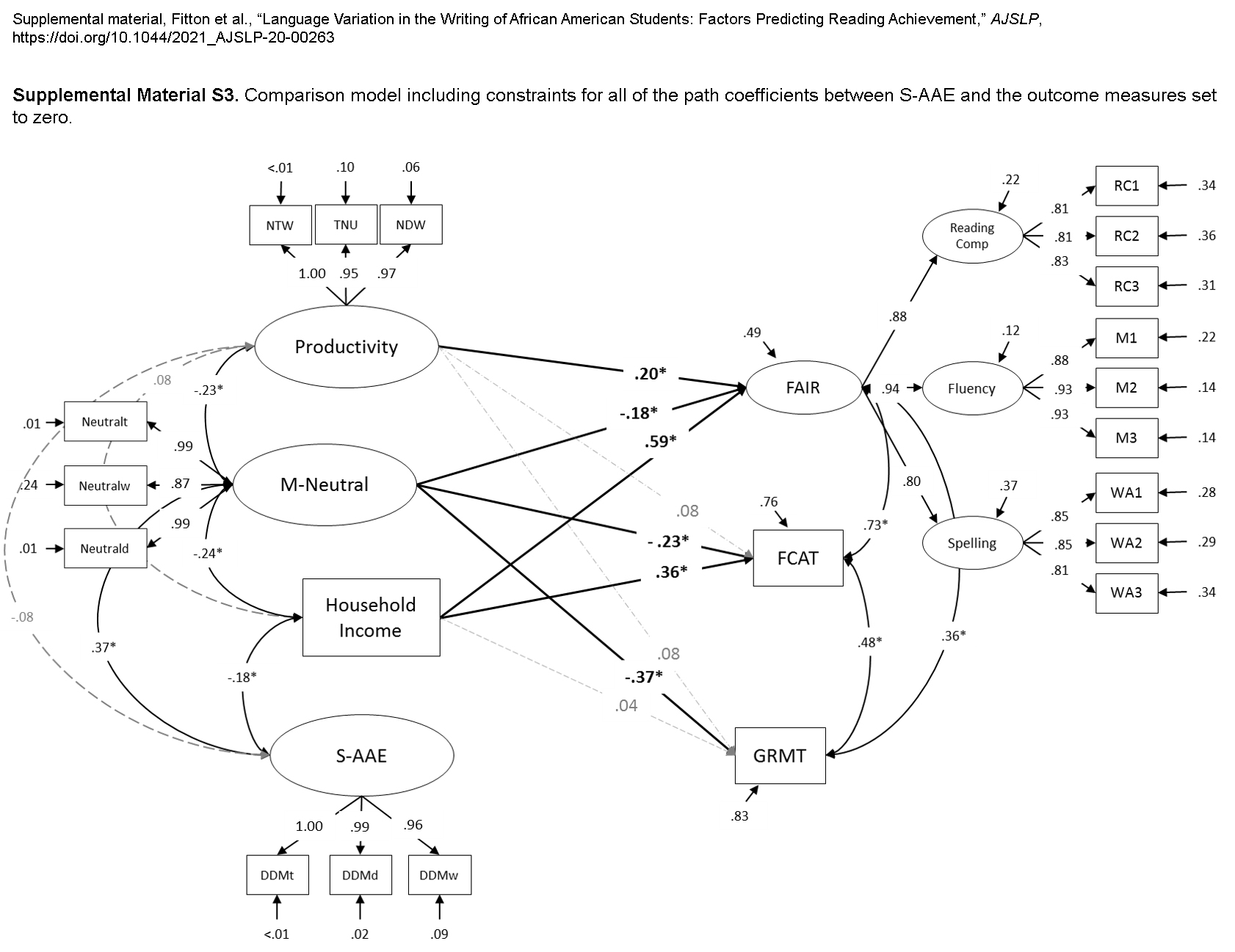

Supplement: Supplemental Material S3 [file AJSLP-30-2653-s003.jpg]
